# Uso de Estatinas Melhora a Proteção Cardiometabólica Promovida pelo Treinamento Físico em Ambiente Aquático: Um Estudo Clínico Randomizado

**DOI:** 10.36660/abc.20210746

**Published:** 2022-05-04

**Authors:** Carla Paixão Miranda, Fernando Botoni, Manoel Rocha

**Affiliations:** 1 Universidade de Brasília Brasília DF Brasil Universidade de Brasília - Patologia Molecular,Brasília, DF – Brasil; 2 Universidade Federal de Minas Gerais Belo Horizonte MG Brasil Universidade Federal de Minas Gerais, Belo Horizonte, MG – Brasil

**Keywords:** Estatinas, Exercício, Atividade Física, Exercício Aquático, Hipertensão, Diabetes Mellitus, Síndrome Metabólica

**Prezado editor,**

Li com interesse o artigo publicado por Costa et al.,^1^ O uso de estatinas na proteção cardiometabólica promovida por treinamento físico de intensidade moderada em ambiente aquático, pouco tem sido estudado principalmente sob o aspecto clínico. No entanto, como é descrito por Costa et al.,^1^ o treinamento de força em ambiente aquático aliado ao uso de estatinas tem sido um incremento efetivo por promover adaptações metabólicas e redução dos níveis lipídicos. Os preditores prognósticos associados ao risco de morte por doença cardiovascular foram medidos nos três grupos: treinamento em meio aquático (WA), treinamento de força (WR) e grupo controle (GC). No entanto, um grupo controle saudável não foi acrescido no estudo e por isso o poder estatístico do teste poderia ter sido maior. Segundo análise do índice de massa corporal (IMC) do grupo (GC) os participantes eram obesos, não sendo isto explicado nos critérios de inclusão. Além disso, não houve homogeneidade no número de indivíduos com medicação (MED) e não medicados (NMED). O indicador de intensidade do treinamento aeróbico, se for relacionado com outras variáveis poderá acrescentar novas perguntas e linhas de pensamentos e pesquisas. O efeito da estatina tem sido investigado na função do musculo esquelético, na performance e na capacidade funcional de atletas em diferentes modalidades esportivas e de intensidade.^2 , 3^ Estudo randomizado duplo cego mostrou o efeito protetor da estatina na redução dos níveis de citocinas pró-inflamatórias com aumento nas concentrações médias da creatina-fosfoquinase (PCK), essa enzima desempenha importante papel regulador no metabolismo intracelular, dos tecidos contrateis, nos músculos estriados esqueléticos, tecido cardíaco e cérebro.^4^ Por tanto, no estudo de Costa et al.,^1^ as conclusões respondem ao objetivo proposto e suas fundamentações teóricas estão alinhadas à pergunta e à hipótese do estudo.

## Carta-resposta

Em 29 de setembro de 2021, recebemos a notificação de uma carta ao editor da revista Arquivos Brasileiros de Cardiologia que manifestavam algumas críticas ao artigo de nossa autoria, intitulado “Uso de estatinas melhora a proteção cardiometabólica promovida pelo treinamento físico em ambiente aquático: um ensaio clínico randomizado”.^1^ Agradecemos a oportunidade de resposta dada pelos editores e gostaríamos de esclarecer alguns pontos apontados no documento enviado. Entendemos que ela apresenta problemas graves de interpretação dos processos de metodologia científica empregados em nosso artigo. Os autores da missiva relatam que um grupo controle saudável não foi adicionado ao trabalho – o que é verdade. A seguir, relatamos os motivos pelos quais tomamos esta decisão metodológica.

O objetivo do estudo foi analisar a influência do uso de sinvastatina nas adaptações do perfil lipídico, decorrentes do treinamento aeróbico em meio aquático e da resistência em mulheres idosas com dislipidemia. Considerando que a literatura é vasta em estudos de intervenções de treinamento físico com a população saudável e em avaliação de parâmetros lipídicos,^2^ o foco do nosso experimento foi estudar os efeitos em pessoas com dislipidemia, trazendo um resultado novo e focado em quem mais necessita de intervenções terapêuticas, visando à melhora do perfil lipídico. Nesse contexto, não visualizamos justificativa para inserir um grupo controle saudável. Outra razão é a ausência na intenção de comparar os efeitos do uso de uma medicação hipolipemiante em indivíduos saudáveis em relação aos pacientes com dislipidemia. Dessa forma, não encontramos quaisquer justificativas para utilizar essa medicação na população saudável.

Na carta, também é relatado que o poder estatístico do teste realizado poderia ter sido maior. Entendemos que, embora não tenhamos apresentado os resultados do poder estatístico, a apuração está adequada, considerando que as análises foram realizadas em tamanho superior ao solicitado pelo cálculo do tamanho de amostra. Além disso, foram respeitados todos os pressupostos que envolvem as equações de estimativas generalizadas.^3^ Cabe ressaltar que se realizou o cálculo do tamanho da amostra conforme sugerido nas normas da metodologia científica *a priori* , ou seja, na fase de projeto de pesquisa, previamente à coleta de dados. Logo, levaram-se em consideração tópicos como a análise estatística prevista, a variabilidade do desfecho e o delineamento da pesquisa.^4 - 6^

Ademais, os autores da carta levantam a questão de que o índice de massa corporal (IMC) do grupo controle os caracteriza como obesos e que isso não foi explicado nos critérios de inclusão. De fato, o IMC do estrato citado classifica os participantes dessa forma, de acordo com os critérios da Organização Mundial da Saúde (OMS), mas essa é apenas uma característica do grupo, e não um critério de inclusão do trabalho, como pode ser visualizado no capítulo de métodos (subcapítulo de participantes e critérios de elegibilidade).

Os autores da carta mencionam que não houve homogeneidade no número de indivíduos com medicação (MED) e sem medicação (NMED). No entanto, é possível visualizar na Tabela 1 de nosso artigo que houve um equilíbrio na distribuição de usuários e não usuários de estatinas entre os três grupos do estudo, sendo dez usuários no grupo aeróbio, nove no grupo força e nove no grupo controle, não havendo diferença estatística na distribuição entre os grupos (p = 0,639). Da mesma maneira, não houve diferença na distribuição de usuários de 20 mg de estatinas entre os grupos (p = 0,961) nem de 40 mg de estatinas entre os grupos (p = 0,961) (Tabela 1 do artigo).

Os autores da carta também relatam que “o indicador de intensidade do treinamento aeróbio, se relacionado com outras variáveis poderão acrescentar novas perguntas que significam linhas de pensamentos e pesquisas que este estudo descortina, haja vista que o efeito da estatina tem sido investigado na função do músculo esquelético, performance e na capacidade funcional de atletas em diferentes modalidades esportivas e de intensidade”. Cabe ressaltar que o parâmetro utilizado para prescrição da intensidade do treinamento aeróbico (frequência cardíaca relativa ao limiar anaeróbio) é considerado há algumas décadas o método mais robusto, conforme indicado na literatura.^7^ Tal fato possibilita estimar os limites das zonas de treinamento aeróbico e anaeróbio. Entendemos isso como um ponto positivo do estudo e que não se relaciona com os já conhecidos efeitos das estatinas na função musculoesquelética. À luz do nosso conhecimento, independentemente do parâmetro utilizado para a prescrição do treinamento aeróbio, os efeitos deverão ser os mesmos.

Por fim, agradecemos o reconhecimento de que as conclusões de nosso estudo respondem ao objetivo proposto e que as fundamentações teóricas estão alinhadas à pergunta e a hipótese dele.

**Rochelle Costa**

**Alexandra Vieira**

**Leandro Coconcelli**

**Alex Fagundes**

**Adriana Cristine Buttelli**

**Laura Pereira**

**Ricardo Stein**

**Luiz Fernando Kruel**
