# Supplementary material for: Uso de Estatinas Melhora a Proteção Cardiometabólica Promovida pelo Treinamento Físico em Ambiente Aquático: Um Estudo Clínico Randomizado
Source: Arq Bras Cardiol. 2022 May 4;118(5):1002–4. [Article in Portuguese] doi: 10.36660/abc.20210746 (PMC9368874; doi:10.36660/abc.20210746)
Supplement: Translation

# Translation: Statin Use Improves Cardiometabolic Protection Promoted By Physical Training in an Aquatic Environment: A Randomized Clinical Trial

**Authors:** Carla Paixão Miranda, Fernando Botoni, Manoel Rocha

**Affiliations:** 1 Brasil Universidade de Brasília - Patologia Molecular,Brasília, DF – Brasil; 2 Brasil Universidade Federal de Minas Gerais, Belo Horizonte, MG – Brasil

**Keywords:** Statins, Exercise, Physical Activity, Aquatic Environment, Hypertension, Diabetes Mellitus, Metabolica Syndome/complications

**Dear editor,**

I read with interest the article published by Costa et al.^1^ The use of statins in cardiometabolic protection promoted by physical training of moderate-intensity in an aquatic environment has been little studied, mainly from the clinical point of view. However, as described by Costa et al.,^1^ strength training in an aquatic environment combined with statins has been an effective increase in promoting metabolic adaptations and a reduction in lipid levels. The prognostic predictors associated with the risk of death from cardiovascular disease were measured in the three groups: aquatic training (AT), strength training (ST) and control group (CG). However, a healthy control group was not added to the study, and therefore the statistical power of the test could have been greater. According to the analysis of the body mass index (BMI) of the group (CG), the participants were obese, which was not explained in the inclusion criteria. In addition, there was no homogeneity in the number of individuals on medication (MED) and not on medication (NMED). If related to other variables, the aerobic training intensity indicator may add new questions and lines of thought and research. The effect of statins has been investigated on skeletal muscle function, performance and functional capacity of athletes in different sports and intensity modalities.^2 , 3^ A randomized, double-blind study showed the protective effect of a statin in reducing the levels of pro-inflammatory cytokines with an increase in the mean concentrations of creatine phosphokinase (PCK); this enzyme plays an important regulatory role in intracellular metabolism, in contractile tissues, in skeletal striated muscles, heart tissue and brain.^4^ Therefore, in the study by Costa et al.,^1^ the conclusions respond to the proposed objective, and its theoretical foundations are in line with the question and hypothesis of the study.

## Reply

On 09/29/21 we received notification of a Letter to the Editor of the *Arquivos Brasileiros de Cardiologia* journal with some criticisms to our article entitled “Statin Use Improves Cardiometabolic Protection Promoted By Physical Training in an Aquatic Environment: A Randomized Clinical Trial”.^1^ We would like to thank the editors for the opportunity to respond and would like to clarify some points mentioned in the aforementioned Letter, which, according to our understanding, has serious problems in the interpretation of the scientific methodology processes used in our article.

The authors of the Letter report that a healthy control group was not added to the study. In fact, it was not and we report below the reasons why we made this methodological decision. The objective of the study was to analyze the influence of simvastatin use on the lipid profile adaptations resulting from aerobic and resistance training in aquatic environments in elderly women with dyslipidemia. Considering that the literature is already vast concerning studies of physical training interventions with a healthy population and evaluation of lipid parameters,^2^ the focus of our experiment was to study their effects on individuals with dyslipidemia, thus bringing a new result focused on those who need therapeutic interventions the most, aiming at improving the lipid profile. In this context, we did not see the justification for the inclusion of a healthy group. Another reason is the complete lack of intention to compare the effects of using a lipid-lowering medication in healthy individuals *versus* dyslipidemia patients. Therefore, we did not find any justification for the use of this medication in a healthy population.

The Letter also mentioned that the statistical power of the performed test could have been greater. We understand that, although we did not disclose the results of statistical power, it is appropriate, considering that the analyses were performed with a sample size larger than that estimated by the sample size calculation. In addition, all assumptions involving the generalized estimating equations^3^ were followed. It should be noted that the sample size calculation was performed as suggested in the rules of scientific methodology *a priori* , that is, in the research project phase, prior to data collection. Therefore, this calculation took into account topics such as predicted statistical analysis, outcome variability and research design.^4 - 6^

Moreover, the authors of the Letter raise the issue that the body mass index (BMI) of the control group characterizes them as obese and this was not explained in the inclusion criteria. In fact, the BMI of the control group classifies the participants as obese, according to the World Health Organization criteria, but this is just a characteristic of the group, and it was not an inclusion criterion of the study, as shown in the methods section (subsection on participants and eligibility criteria).

The authors of the Letter mention that there was no homogeneity in the number of individuals on medication (MED) and without medication (NMED). However, it is possible to see in Table 1 of our article that there was a balance in the distribution of users and non-users of statins between the three study groups, with 10 users in the aerobic group, 9 in the resistance training group and 9 in the control group, with no statistical difference in the distribution between the groups (p=0.639). Likewise, there was no difference in the distribution of users of 20mg of statins between the groups (p=0.961) or of 40mg of statins between the groups (p=0.961) (Table 1).

Finally, the authors of the Letter report that: “The aerobic training intensity indicator, when related to other variables, may add new questions that indicate lines of thought and research that this study discloses, given that the statin effect has been investigated on the skeletal muscle function, performance and functional capacity of athletes in different sports modalities and intensity”. It should be noted that the parameter used to prescribe the aerobic training intensity (heart rate relative to the anaerobic threshold) has been considered, for some decades, the most robust method, as indicated in the literature,^7^ which allows estimating the thresholds of aerobic and anaerobic training zones. We understand this as a positive point of the study and that it is not related to the already known effects of statins on musculoskeletal function. In the light of our knowledge, regardless of the parameter used for aerobic training prescription, the effects of statins on musculoskeletal function should be the same.

Finally, we are grateful for the acknowledgement that the conclusions of our study answer the proposed objective and that the theoretical foundations are in line with the study question and hypothesis.

**Rochelle Costa**

**Alexandra Vieira**

**Leandro Coconcelli**

**Alex Fagundes**

**Adriana Cristine Buttelli**

**Laura Pereira**

**Ricardo Stein**

**Luiz Fernando Kruel**
